# Sarcoidosis

**DOI:** 10.1186/1750-1172-2-46

**Published:** 2007-11-19

**Authors:** Hilario Nunes, Diane Bouvry, Paul Soler, Dominique Valeyre

**Affiliations:** 1 Service de Pneumologie, Hôpital Avicenne, Assistance Publique Hôpitaux de Paris et Faculté de Médecine, Université Paris-Nord, 93009 Bobigny, France; 2 Inserm U700, Faculté de Médecine Paris VII, site Xavier Bichat, 75018 Paris, France

## Abstract

Sarcoidosis is a multisystemic disorder of unknown cause characterized by the formation of immune granulomas in involved organs. It is an ubiquitous disease with incidence (varying according to age, sex, race and geographic origin) estimated at around 16.5/100,000 in men and 19/100,000 in women. The lung and the lymphatic system are predominantly affected but virtually every organ may be involved. Other severe manifestations result from cardiac, neurological, ocular, kidney or laryngeal localizations. In most cases, sarcoidosis is revealed by persistent dry cough, eye or skin manifestations, peripheral lymph nodes, fatigue, weight loss, fever or night sweats, and erythema nodosum. Abnormal metabolism of vitamin D3 within granulomatous lesions and hypercalcemia are possible. Chest radiography is abnormal in about 90% of cases and shows lymphadenopathy and/or pulmonary infiltrates (without or with fibrosis), defining sarcoidosis stages from I to IV. The etiology remains unknown but the prevailing hypothesis is that various unidentified, likely poorly degradable antigens of either infectious or environmental origin could trigger an exaggerated immune reaction in genetically susceptible hosts. Diagnosis relies on compatible clinical and radiographic manifestations, evidence of non-caseating granulomas obtained by biopsy through tracheobronchial endoscopy or at other sites, and exclusion of all other granulomatous diseases. The evolution and severity of sarcoidosis are highly variable. Mortality is estimated at between 0.5–5%. In most benign cases (spontaneous resolution within 24–36 months), no treatment is required but a regular follow-up until recovery is necessary. In more serious cases, a medical treatment has to be prescribed either initially or at some point during follow-up according to clinical manifestations and their evolution. Systemic corticosteroids are the mainstay of treatment of sarcoidosis. The minimal duration of treatment is 12 months. Some patients experience repeated relapses and may require long-term low-dose corticosteroid therapy during years. Other treatments (immunosuppressive drugs and aminoquinolins) may be useful in case of unsatisfactory response to corticosteroids, poor tolerance and as sparing agents when high doses of corticosteroids are needed for a long time. In some strictly selected cases refractory to standard therapy, specific antiTNF-**α **agents may offer precious improvement. Some patients benefit from topical corticosteroids.

## Disease name

Sarcoidosis

## Definition

Sarcoidosis is a multisystemic disorder of unknown cause that is characterized by the formation of immune granulomas in involved organs [1-4].

## Background

Sarcoidosis affects primarily young and middle-aged adults. A prevailing hypothesis is that various unidentified antigens, either infectious or environnemental, could trigger an exaggerated immune reaction in genetically susceptible hosts [2]. The clinical expression of sarcoidosis is protean, in particular as regards the number and sites of involved organs. The lung and the lymphatic system are predominantly affected but virtually virtually every organ may be affected. Skin, eyes, peripheral lymph nodes and liver are each involved in 10–25% of cases. The evolution and severity of the disease are highly variable. Whilst spontaneous resolution occurs in the majority of cases within 12–36 months, some patients experience a prolonged and serious course [3]. Pulmonary fibrosis is the most frequent severe manifestation, accounting for the major cause of morbidity and mortality in western countries [3]. Other severe manifestations of the disease consist of cardiac, neurological, ocular, kidney or laryngeal localizations. Sarcoidosis can also induce abnormal metabolism of vitamin D3 within granulomatous lesions and hypercalcemia [5]. Mortality attributable to sarcoidosis is estimated at between 0.5 and 5% [6] and results from lung, heart and central nervous system localizations [3].

Although not always required, the mainstay of sarcoidosis treatment is systemic corticosteroids [7,8]. Corticosteroids impeed the formation of granulomas and, as a result, are largely efficient against most active clinical manifestations. However, treatment is merely suspensive with frequent relapses when withdrawal is too rapid. Furthermore, the adverse side effects of corticosteroids are substantial. Other immunosuppressive agents and aminoquinolins can be employed as sparing drugs or as alternative options to corticosteroid therapy in patients with unsatisfactory response or poor tolerance with corticosteroids, or when high doses of corticosteroids are needed for a long time [8]. Recent advances on the knowledge of sarcoidosis pathogeny suggest that the overproduction of tumor necrosis factor **α **(TNF-**α**) at the sites of disease plays a pivotal role [2]. Interstingly, most agents that are effective in sarcoidosis also appear to have antiTNF-**α **properties, either specifically or as part of broad effects [4].

## Epidemiology

Sarcoidosis is an ubiquitary disease with an incidence varying according to age, sex, race and geographic origin [1,3,4,9]. Sarcoidosis incidence is globally estimated at around 16.5/100,000 in men and 19/100,000 in women [10]. The lifetime incidence is higher in women (1.3%) than in men (1%), and in Blacks (2.4%) than in Caucasians (0.8%) [10,11]. Although possible at all ages, a predilection is observed between 25 and 40 years in both genders at least in Scandinavian countries and Japan [3,10], and a second peak of incidence has been reported in women over 50 years of age in some but not all published series [10]. The incidence of sarcoidosis is remarkably low before 15 years (1/100,000) and exceptional before 4 years (0.06/100,000) [12]. The clinical expression and severity of sarcoidosis depend upon epidemiological factors. Erythema nodosum is seen in 3–44% of cases but it is far more frequent in women than in men, in Caucasians than in Blacks or in Japanese and in northern than in southern european countries [1,3,12]. In Blacks, the disease is more likely to be disseminated, showing a higher frequency of ophtalmological, cutaneous, hepatic and lymphatic localizations [9] and it is undeniably more severe [3]. In Japanese patients, cardiac and ophthalmological involvement is common [3]. Sarcoidosis is generally sporadic but a familial aggregation is found in 1.7–17% of cases [12,13]. The risk of sarcoidosis is increased in the family of an index case with siblings having the highest relative risk (odd ratio = 5.8) [13]. Interestingly, there are strands of evidence provided by familial sarcoidosis supporting the role of genetic factors: the similarity of phenotypical presentation among families, the similarity of the mother/children and father/children ratios, and the higher incidence and phenotypical resemblance between homozygotic twins than between heterozygotic twins.

Time and space-related clusters, tracing case contacts and cases of possible transmission of sarcoidosis *via *transplantation all suggest the role of transmissible agents either of infectious or of environnemental origin, although not identified yet [14].

## Cause and mechanism of sarcoidosis

Sarcoidosis is considered as the consequence of a chronic immunological response associating a genetic susceptibility and specific infectious or environmental factors [15-17]. Sarcoid granuloma, the typical elementary microscopical lesion, is an immune granuloma resulting from a specific cell-mediated immune response to a likely poorly degradable antigenic agent. Numerous cytokines and other mediators are produced by both activated macrophages and T lymphocytes during granulomatous responses. A number of data suggest that interferon gamma (IFN-γ) and cytokines such as TNF-**α**, IL-12 and IL-18 play a critical role in the formation of granulomatous lesions [15]. Pathological similarities between sarcoidosis and tuberculosis have led for a long time to the suggestion that mycobacteria are the cause of sarcoidosis [16]. However, the studies attempting to isolate mycobacteria in sarcoid tissues, even those using the most sensitive polymerase chain reaction (PCR) techniques, failed to detect mycobacterial DNA. *Propiniobacterium acnes *has also been incriminated as a causative agent but its role has not yet been ascertained. Finally, it has been shown that the incidence of sarcoidosis is influenced by genetic susceptibilities and particularly by polymorphisms involving immunity [17]. It seems very probable that genetic factors have also an important effect on disease presentation, progression and overall prognosis [3,17].

No cause of sarcoidosis has yet been comfirmed, despite numerous searches using most emerging diagnostic tools. Several reasons could explain the difficulty to determine an etiology in such a context [16]. First, sarcoidosis may well not be a unique disease with a unique cause. Second, the causal agent may be a yet unidentified microorganism. Last, the mechanism underlying sarcoidosis may result not only from a single organism or antigen but also be related to the presence of adjuvant factors.

## Clinical presentation

The presentation of sarcoidosis depends on epidemiological factors such as age, sex and race, the duration of the disease and the sites of involvement. Asymptomatic presentations, erythema nodosum and hypercalcemia are more frequent in Europeans, while symptomatic and multivisceral presentations are more frequent in Afro-Americans [1-4,9]. Overall, sarcoidosis is mostly revealed in the following circumstances: (i) respiratory symptoms, firstly persistant dry cough in around 30% cases, (ii) extrathoracic localizations, mainly peripheral lymph nodes, eyes or skin, (iii) constitutional symptoms such as fatigue (27%), weight loss (28%), fever (10–17%) or night sweats and (iv) erythema nodosum (3–44%) [1,3,4,18]. Erythema nodosum is usually associated with bilateral intrathoracic lymphadenopathies defining "Löfgren's syndrome" which is a benign form of the disease. Constitutional symptoms, particularly asthenia, are often present and can be a disabling problem [18]. Finally, an incidental discovery of sarcoidosis in asymptomatic patients with chest X-ray aberrations is not uncommon (8–60%) [19].

### Intrathoracic manifestations

Detailed occupational and environmental history is usually unremarkable and exposure to beryllium dusts and to drugs inducing granulomatosis must be excluded. As a general rule, no abnormality is audible at chest physical examination. By contrast, chest X-ray is abnormal at some point in 86–92% of cases and remains a key investigation for diagnosis [1,2,9,19,20]. Radiographic staging of sarcoidosis is based on the presence of lymphadenopathies and lung infiltration without or with fibrosis [3]. Lymphadenopathies are typically hilar, bilateral, symmetrical and non compressive, and often associated with right paratracheal and aortic-pulmonic window lymph node involvement [19,20]. Lung infiltration is usually bilateral, symmetrical and diffuse but with a patent predominance for central regions and upper lobes. The pattern of infiltration is typically micronodular (diffuse punctiform opacities).

Stage I, the most frequent presentation, is defined as isolated intrathoracic lymphadenopathy, stage II as lymphadenopathy accompanied by lung infiltration and stage III as lone parenchymal infiltration. Stage IV refers to overt lung fibrosis. Typical stage I and II are highly reliable for diagnosing sarcoidosis, while stage III and IV are far less accurate [3]. This classification was established more than four decades ago but it still represents a major determinant of prognosis [19]. The probability of further spontaneous healing decreases as a function of initial radiographic stage (55–90% in stage I; 40–70% in stage II; 10–20% in stage III and 0% in stage IV) [3].

Pulmonary function tests typically demonstrate decreased volumes and CO diffusing capacity with functional alteration tending to be more frequent and marked from stage I to stage IV [20]. Using the criterion of forced expiratory volume in 1 sec (FEV1)/vital capacity (VC) ratio < 70%, airway obstruction is encountered in 5.7% of cases [21]. Airway obstruction has been recognized as a marker of poor prognosis including increased morbidity, higher frequency of respiratory symptoms and radiographic stage IV [22,23].

Fiberoptic bronchoscopy yields granulomas by means of mucosal or transbronchial biopsy in 57–88% of cases [3,24,25]. Lymphocytosis in broncho-alveolar lavage (BAL) is observed in 90% of cases and a CD4+/CD8+ T lymphocyte ratio greater than 3.5 in half cases [24]. Transbronchial needle aspiration makes also possible valuable samplings of hilar and mediastinal lymph nodes [25].

Chest high resolution computed tomography (HRCT) has a better diagnosis accuracy than chest X-ray [19,20]. The hallmark of pulmonary sarcoidosis are widespread micronodules with a typical perilymphangitic distribution and a predominance for the middle and upper parts of the lungs. However, HRCT is not always necessary when a confident diagnosis can be made from typical clinical and radiographic features. By contrast, HRCT makes compelling diagnostic contributions in tricky cases and in detecting complications of the lung disease. HRCT is also particularly useful in cases difficult to treat [26].

### Extrathoracic manifestations

Apart from intrathoracic lymph nodes and lung, the most frequent sites of sarcoidosis involvement include peripheral lymph nodes, eyes, skin and liver, each being noted in about 10–25% of cases in most series [1-4,9]. Virtually any organ may be affected by sarcoidosis but the frequency and degree of impairement is variable according to localizations. Overall, extrathoracic manifestations occur in about half of the cases and in this setting are associated with intrathoracic involvement in 80–90% cases. Extrathoracic localizations can be confined to one organ or be multiple and diversely combined.

**Peripheral lymphadenopathies **are easily palpable and their frequency varies between series up to 70%. They are asymptomatic, firm, of various size and every site of lymph nodes may be affected. Peripheral lymphadenopathies are readily accessible to biopsy. They can be localized in the abdomen where they are recognizable by abdominal echography, computed tomography (CT) or magnetic resonance imaging (MRI) and they can be associated with liver or splenic nodules or enlargement.

The frequency of **ocular sarcoidosis **is comprised between 10 and 50% according to published studies. This wide range is due to recruitment bias in series reported by ophtalmologists and to epidemiological factors with an increased incidence in Japanese patients [27]. Any part of the eye may be involved in sarcoidosis. Macroscopic nodules of the conjunctiva are seen in 6–40% of cases and allow evidence of granulomas in 67% of cases [27]. Anterior uveitis can be an initial, acute and symptomatic (red eyes, photophobia and blurred vision) manifestation but it can also be asymptomatic and have a chronic course justifying a systematic eye investigation including a slit lamp examination. Similarly, intermediate uveitis can be symptomatic or not. Posterior uveitis is encountered in up to 28% of cases with eye sarcoidosis and it may be associated with neurologic involvement. Optic neuropathy is very rare but it may provoke a rapid and definitive loss of vision in the absence of immediate and adequate systemic treatment. Lacrimal involvement may lead to sicca keratoconjonctivitis, while bilateral enlargement of lacrimal gland is unfrequent.

**Skin manifestations **of sarcoidosis are heterogeneous. Erythema nodosum is a non specific association of sarcoidosis realizing typically Löfgren's syndrome in the presence of bilateral hilar lymphadenopathy. The incidence of Löfgren's syndrome varies according to epidemiological factors (see "Epidemiology"). The frequency of specific skin manifestations of sarcoidosis ranges from 10 to 40% cases [28]. They appear at any stage of the disease and can remain strictly isolated in one third of cases [28]. The clinical picture of skin sarcoidosis is variegated: maculopapular lesions of various size, changes of old scars, lupus pernio, plaque formation, subcutaneous lesions *etc*. Skin lesions supply a plain and proper site for biopsy with the exception of erythema nodosum. Lesions of the face such as lupus pernio are very unpleasant and often linked with a longstanding evolution of sarcoidosis and osseous and sinonasal localizations. They are often difficult to control with treatments.

#### Liver involvement

while granulomas are found in up to 60–80% of liver biopsy specimens, abnormalities of biological tests, primarily cholestasis, are evidenced in only about 20% of cases. Clinical enlargement of the liver is far less frequent. Chronic intrahepatic cholestasis, hepatic dysfunction, cirrhosis and portal hypertension are all severe but rare complications of sarcoidosis.

Localizations of sarcoidosis in heart, central nervous system, larynx or kidney are less frequent but potentially serious.

**Cardiac involvement **of sarcoidosis appears to be much more frequent in Japanese population, particularly in females > 50 years, than in Europeans and Americans in which it concerns approximatively 5% of cases [29,30]. It can occur at any point of time during the course of sarcoidosis. The left ventricular myocardium and, more specifically, the interventricular septum and the free left lateral wall are the most frequently involved structures in sarcoidosis. Main relevant signs include atrio-ventricular block, complete right bundle branch block (which is notably frequent and suggestive), ventricular hyperexcitability, ventricular tachycardia, left ventricular dysfunction and sudden death. The diagnosis of cardiac sarcoidosis is often a challenging issue for physicians. Serial electrocardiogram (ECG) during evolution survey, echocardiography, 24-h Holter monitoring of ECG, Thallium scan, MRI and and ^18^FDG PET can be helpful tools for diagnosis (29,30). Endomyocardial biopsy is theoretically the most confident mean to ascertain the diagnosis but in clinical practice it lacks sensitivity and it is an invasive procedure, which constitutes major limitations. Thus, the diagnosis of cardiac sarcoidosis usually relies on the conjunction of multiple arguments: (i) evidence of sarcoidosis, (ii) presence of cardiac abnormalities compatible with cardiac sarcoidosis and (iii) exclusion of any other cause of cardiac disease.

Any part of the **nervous system **can be involved in sarcoidosis with a frequency around 10% [31,32]: meninges, central nervous system, cranial nerves and peripheral nerves. Aseptic meningitis can cause symptoms such as fever or headache and be associated with central nervous system manifestations or cranial neuropathy but can also be asymptomatic. Central nervous system manifestations are most frequent in Caucasians. Various clinical expression can be observed: neuro-endocrine symptoms, psychiatric symptoms, seizures, cognitive abnormalities, hydrocephalia, spinal cord impairment and various neurologic deficits. Brain and spinal cord MRI are the most sensitive tests to diagnose central nervous system sarcoidosis and guide therapeutical management. Cranial neuropathies prevale in Black patients. Although all cranial nerves can be concerned, seventh nerve palsy is the most common sign followed by optic neuropathy and involvement of the eighth and fifth nerves. Heerfordt's syndrome which associates uveitis, parotid gland enlargement, fever and cranial neuropathy, usually seventh nerve palsy, is highly suggestive of sarcoidosis. The diagnosis of neurosarcoidosis relies on the conjunction of: (i) confirmed sarcoidosis, (ii) neurologic involvement compatible with neurosarcoidosis and (iii) exclusion of an alternative neurologic disorder.

Clinically significant involvement of **kidneys **is extremely rare, cited in 0.7% of cases [9,33]. Histology typically reveals granulomatous interstitial nephritis. Biology shows decreased creatinine clearance and low or absent proteinuria. Sarcoidosis can also produce urinary lithiasis and nephrocalcinosis while the relations between diverse forms of glomerulonephritis which are very uncommon and sarcoidoisis are very unclear.

**Parotid **enlargement is seen in 5–10% of cases. Sarcoidosis of the **upper respiratory tract **occurrs in 0.7 and 6 % and may assume various features in relation to the involvement of sinonasal mucosa, pharynx and larynx. Laryngeal sarcoidosis is potentially serious by provoking airway obstruction. Sinonasal sarcoidosis is well-recognised to be a chronic and recalcitrant form of the disease, which is associated with lupus pernio in half cases [34,35]. Typically, patients complain of chronic crusting rhinitis but sinonasal involvement can occasionally lead to bone lysis and eventually disfiguiring saddle nose [35]. **Articular **involvement may be acute and transient or chronic and persistent [36]. Yet, whilst joint pains occur in 25–39% of patients with sarcoidosis, deforming arthritis is rare. **Osseous **involvement is associated with characteristic abnormalities on radiography [36]. Sarcoidosis usually affects small joints of the hands and feet, knees, ankles, elbows and wrists. Sympomatic muscle involvement is particularly unfrequent (1.4–2.3% cases). **Gastrointestinal tract **is involved in less than 1.0%. The stomach is the most commonly involved part of gastronintestinal tract, sometimes diagnosed incidentally [37]. Sarcoidosis of the small intestine and colon is much rarer and may mimic Crohn's disease. Pancreas and peritoneal localizations are exceptional. **Splenic **enlargement is observed in 5–10%, it is usually minimal and asymptomatic and causes rarely decrease in the count of platelets, red and white cells [37]. In the absence of splenomegaly, hematological alterations may be exceptionally due to a granulomatous infiltration of the bone marrow or to an autoimmune process, mainly hemolytic anemia or thrombopenia [37]. However, the most frequent abnormality is lymphopenia, which mechanism is a redistribution of blood T cells to sites of disease.

### Biologic manifestations

Hypercalcemia is present in around 11% and hypercalciuria in around 36% of cases [33]. Serum angiotensin converting enzyme (SACE) is believed to reflect disease activity and dissemination but it is increased in about 60% of cases [24]. Serum protein electrophoresis shows polyclonal hypergammaglobulinemia in half cases. Hypogammaglobulinemia must prompt clinicians to seek alternative diagnosis to sarcoidosis, mainly variable common immunodeficiency and lymphoma. Routine blood tests are useful to evidence abnormal hepatic function and more rarely increased creatinine, and hematologic abnormalities, as discussed above.

### Special situations

#### Sarcoidosis and pregnancy

Sarcoidosis does not influence pregnancy adversely. Whether or not the disease improves during pregnancy remains debated. On the other hand, it is clear that sarcoidosis may exacerbate again in the puerperium, which justifies a close surveillance within a period of 6 months after delivery. Pregancy should be avoided when treatments other than corticosteroids are necessary because of potential foetal toxicity or teratogenicity [37]. It is also contra-indicated in case of severe visceral involvement, particularly advanced respiratory insufficiency.

#### Sarcoidosis in children

Sarcoidosis is rare before the age of 15 and exceptional before 4. The disease of chidren resembles that of adults with respect to the distribution of organs involved. In old literature, sarcoidosis with an onset in children under the age of 4 has been reported to have an original presentation characterized by the combination of polyarthritis, uveitis and skin rash, and the rarity of intrathoracic involvement. It is likely that most of reported cases had actually Blau syndrome.

### Association of sarcoidosis with other conditions

Co-existence of sarcoidosis with diverse autoimmune conditions, other granulomatosis and proliferations in the same individual is classical, even though still controversial. These include all connective tissue diseases but principally scleroderma and Sjogren's syndrome, as well as ankylosing spondilitis, primary biliary cirrhosis and auto-immune disorders of the thyroid [37]. Interestingly, an association with Crohn's disease, another granulomatous disease of unknown cause, has been reported [37]. Finally, association of lymphoproliferative and solid malignancies must be kept in mind and can raise difficult diagnosis problems.

## Diagnosis

The diagnosis of sarcoidosis relies on clinico-radiographical presentation and evidence of non caseating granulomas on biopsy specimens after appropriate exclusion of other granulomatous disorders [3,24]. Diagnosis may benefit from the amalgamation of other supporting arguments such as typical findings on HRCT, CD4/CD8 T lymphocyte ratio higher than 3.5 in BAL, SACE level twice higher than the upper normal limit or typical abnormal calcium metabolism.

Most recommended sites to obtain a histological confirmation are mucosal or transbronchial biopsy through fiberoptic bronchoscopy [3,24], skin lesions, peripheral lymphadenopathy, conjonctival nodules or lip biopsy of accessory salivary glands. Sampling hilar or mediastinal lymph nodes through transbronchial needle aspiration or mediastinoscopy may be required [3,24,25]. Videothoracoscopic lung biopsy may rarely be necessary, while other sites can be guided by clinical findings. The diagnostic yield of all these procedures is more than 90% [3]. Some very typical cases like Löfgren's syndrome may obviate an immediate invasive biopsy procedure but histologic confirmation is mandatory in difficult cases or before treatment initiation.

Gallium-67-citrate (^67^Ga) scintigraphy has long been used as a diagnostic tool in sarcoidosis [38] but is only rarely done now. Uptakes of ^67^Ga is seen in intrathoracic and various extrathoracic localizations of the disease. The current indications of ^67^Ga scintigraphy are as follows: (i) to substantiate the diagnosis in difficult cases, particularly in those with normal chest-X ray and a suspicion of sarcoidosis, when the "lambda plus panda" patterns is found, which is almost pathognomonic of the disease, (ii) to identify other clinically silent extra-pulmonary uptakes and provide potential sites for biopsies, (iii) and to assess activity in difficult cases such as stage IV disease in which whether or not to initiate potentially toxic therapy is a dilemma. Recently, an increasing literature on 18F-fluoro-2-deoxyglucose positron emission tomography (^18^FDG PET) in sarcoidosis has been published, but its role remains to be delineated [38]. Moreover, one should bear in mind the high level of radiation dose delivered by these isotopic investigations.

## Differential diagnosis

Other granulomatous diseases need to be ruled out before the diagnosis of sarcoidosis is ascertained [3,24]. In almost all cases, clinical history, epidemiology with an appropriate investigation of exposure to infections such as tuberculosis or to beryllium inhalation, together with a proper evaluation of all tests are sufficient to discriminate between sarcoidosis and other granulomatous disorders. Tuberculosis, some fungal infections, chronic beryllium disease, granulomatous disorder induced by interferon **α **and **β **or intravesical BCG therapy or associated with common variable immunodeficiency are those conditions which can best mimic sarcoidosis. In infants under four years old, the Blau syndrome has to be considered before sarcoidosis, specially in the absence of thoracic involvement. Loco-regional granulomatous reactions satellite of breast or lung carcinoma and of lymphomas are rarely difficult to differenciate from sarcoidosis as well as other granulomatous disorders of unknown cause like Crohn's disease or Wegener granulomatosis.

## Prognosis and evolution

Sarcoidosis is generally a time-limited disease that lasts for 12–36 months in half cases, for less than 5 years in most remaining cases and occasionnally for multiple decades [3]. Serial assessments are warranted until disease recovery in order to appreciate individual evolution. A minimum delay of three years is necessary after discontinuation of therapy before healing can be established [3].

The most valid prognostic factors remain the mode of onset, the disease extent and chest-X ray staging. An acute onset with Löfgren's syndrome or asymptomatic bilateral hilar lymphadenopathy usually confers a self-limiting course [1-4]. On the other hand, Black race, age of onset > 40 years, lupus pernio, chronic uveitis, sinonasal or osseous localizations, central nervous system or cardiac involvement, chronic hypercalcemia, nephrocalcinosis and stage III and IV are linked to more severe long-standing evolution [3,20,34,35]. Procedures aimed to stage disease activity, particularly gallium scanning and BAL, have been proved to be disappointing in prognosis assessment.

The most severe and frequent complication of sarcoidosis is the occurrence of pulmonary fibrosis. This is usually associated with chronic dyspnea and frank impairment of pulmonary function. Pulmonary fibrosis is the most frequent cause of respiratory failure and results in the majority of deaths related to sarcoidosis in western countries. Pulmonary fibrosis is the consequence of chronic unremitting granulomatous process in the lung. The concern about the development of pulmonary fibrosis is the reason for treating cases of sarcoidosis with persistant pulmonary infiltration.

Airway obstruction can be due to several mechanisms in sarcoidosis: bronchial distortion secondary to pulmonary fibrosis, direct localization of granulomas in the airways, or more seldom extrinsic bronchial compression by hilar or mediastinal lymphadenopathy.

Pulmonary hypertension, *i.e*. increased pulmonary arterial pressure usually occurs in end-stage fibrotic cases. The severity of pulmonary hypertension is often out of proportion to pulmonary function impairement, which implies the role of alternative mechanisms including specific involvement of small lung vessels by sarcoidosis [39]. Pulmonary hypertension is a strong predictive indicator of mortality.

Mycetoma formation is a recognized complication of advanced pulmonary fibrosis and it is believed to be associated with an increased risk of death because of severe bleeding or underlying respiratory insufficiency.

Some other severe localizations may also punctuate the evolution of sarcoidosis. In cardiac sarcoidosis, episodes of sustained ventricular tachycardia coupled with left ventricular dilatation are associated with an increased risk of death [40]. Central nervous system sarcoidosis may represent the third cause of death after lung and cardiac localizions. Various other conditions, even though non lethal, can be associated with important discomfort and disability: disgracious skin manifestations, particularly lupus pernio, sinonasal and laryngeal localizations, renal manifestations and severe ocular localizations. Overall, 10–20% of sarcoidosis patients suffer from permanent sequelae [3]. Treatments which are often given for long periods of time are frequently the source of various troublesome adverse effects. This is particularly the case with corticosteroids that may be responsible for overweight, systemic arterial hypertension, diabetes mellitus, myopathy, buffalo neck *etc*.

## Treatment

The appropriate therapy for sarcoidosis has not yet been well defined for all patients [3,7,9]. Between 30 and 70% of patients never require therapy. In the other cases, a treatment, often corticosteroids, is necessary, either at the onset of the disease or during follow-up because of various consequences.

Cardiac, neurological, renal, ocular sarcoidosis not responding to topical therapy and malignant hypercalcemia always necessitate systemic therapy. Incapacitating general symptoms may lead to short-course corticosteroid therapy [1-4,7,8]. For pulmonary disease, systemic therapy is recommended in patients with stage II or III who are symptomatic or who disclose significant deterioration in serial pulmonary function tests and/or chest X-ray. Treatment is clearly not indicated in asymptomatic stage I. In Löfgren syndrome, erythema nodosum justifies non-steroidal anti-inflammatory drugs or colchicine for some weeks (but rarely corticosteroids) taking into account the usually good prognosis in this setting. Stage IV with advanced pulmonary fibrosis is usually poorly or not at all affected by systemic therapy. However, a trial of therapy to identify residual inflammation is warranted particularly in patients with persistent signs of activity. For pulmonary sarcoidosis, protocols suggest the initial dose of 20 to 40 mg daily of prednisone or its equivalent on alternate days. Higher dosage may be necessary to control cardiac, neurologic, renal, ophtalmologic and laryngeal involvement. After 4 weeks to 3 months therapy, posology is tapered. Therapy must be maintained for at least 12 months [3]. Some patients experience repeated relapses and may require long-term low-dose therapy during years.

Antimalarial drugs are a treatment of choice for mild isolated cutaneous lesions [3,8]. Aminoquinolins are also helpful for hypercalcemia. Methotrexate has been the most extensively used among cytotoxics. Methotrexate is effective in pulmonary and most extra-pulmonary manisfestations, particularly skin, ophthalmic, nervous system and musculoskeletal localizations [8]. Methotrexate-induced hepatotoxicity occurs in about 10% of patients with sarcoidosis treated for more than two years. Azathioprine has clearly proved benefit as a corticosteroid sparing agent. Cyclophosphamide has been occasionally offered in refractory disease with interesting results in cortico-resistant cardiac and neurologic sarcoidosis. Thalidomide has demonstrated efficacy for lupus pernio unresponsive to prior therapy. However, its effect on pulmonary disease is unclear. Moreover, the side-effects of thalidomide can be extremely serious. TNF-**α **inhibitors have led to a dramatic improvement in small series or several case reports with refractory lupus pernio, uveitis or central nervous system localization. Recently, a double-blind study demonstrated a short-timed efficiency of infliximab in severe pulmonary localizations [41]. Yet, etanercept seems less promising as shown in a prospective open-label phase-2 trial. Controlled studies are needed to clearly identify the patients who may profit from these novel therapies.

Currently available agents for the prevention of glucocorticoid-induced osteoporosis may not be safe in sarcoidosis. Vitamin D should be avoided. Calcium supplementation may be given to patients without hypercalciuria under periodic survey of calcium metabolism. Bisphosphonates are also efficient in this setting.

Symptomatic treatments and recommendations may be given in sarcoidosis: low calcium diet and avoidance of sunlight exposure, supplemental oxygen, cardiac drugs, implantable pacemakers or cardiovertible-defibrillator, hormonal substitution, anti-epileptic agents.

Lung, liver and heart transplantation have been successfully performed in patients with end-stage refractory sarcoidosis [42]. Medical therapy must have been exhausted before candidates are selected for transplantation.
